# Toxicity and Antitumor Activity of a Thiophene–Acridine Hybrid

**DOI:** 10.3390/molecules25010064

**Published:** 2019-12-24

**Authors:** Thaís Lisboa, Daiana Silva, Sâmia Duarte, Rafael Ferreira, Camyla Andrade, Ana Luiza Lopes, Juliana Ribeiro, Davi Farias, Ricardo Moura, Malu Reis, Karina Medeiros, Hemerson Magalhães, Marianna Sobral

**Affiliations:** 1 Postgraduate Program in Bioactive Natural and Synthetic Products, Federal University of Paraíba, 58051-970 João Pessoa, Paraíba, Brazil; thaismangeon@gmail.com (T.L.); daiana.frade@gmail.com (D.S.); samiasduarte@gmail.com (S.D.); rafaelcarlos@ltf.ufpb.br (R.F.); camyla.andrade03@gmail.com (C.A.); ana.lopes0407@gmail.com (A.L.L.); hemersoniury@gmail.com (H.M.); 2 Laboratory for Risk Assessment of Novel Technologies, Department of Molecular Biology, Federal University of Paraiba, Campus I, 58051-900 João Pessoa, Brazil; julianaacrs@hotmail.com (J.R.); daviffarias@gmail.com (D.F.); 3 Drug Development and Synthesis Laboratory, Department of Pharmacy, State University of Paraíba, 58070-450 João Pessoa, PB, Brazil; ricardo.olimpiodemoura@gmail.com (R.M.); malureis_farmacia@hotmail.com (M.R.); 4 Department of Morphology, Federal University of Rio Grande do Norte, 59078-970 Natal, RN, Brazil; karinapm@yahoo.com; 5 Department of Pharmaceutical Sciences, Federal University of Paraíba, 58051-970 João Pessoa, Paraíba, Brazil

**Keywords:** colorectal cancer, thiophene–acridine compound, antitumor, cytotoxicity, toxicity

## Abstract

The antitumor effects of thiophene and acridine compounds have been described; however, the clinical usefulness of these compounds is limited due to the risk of high toxicity and drug resistance. The strategy of molecular hybridization presents the opportunity to develop new drugs which may display better target affinity and less serious side effects. Herein, 2-((6-Chloro-2-methoxy-acridin-9-yl)amino)-5,6,7,8-tetrahydro-4H-cyclohepta[b]-thiophene-3-carbonitrile (ACS03), a hybrid thiophene–acridine compound with antileishmanial activity, was tested for toxicity and antitumor activity. The toxicity was evaluated in vitro (on HaCat and peripheral blood mononuclear cells) and in vivo (zebrafish embryos and acute toxicity in mice). Antitumor activity was also assessed in vitro in HCT-116 (human colon carcinoma cell line), K562 (chronic myeloid leukemic cell line), HL-60 (human promyelocytic leukemia cell line), HeLa (human cervical cancer cell line), and MCF-7 (breast cancer cell line) and in vivo (Ehrlich ascites carcinoma model). ACS03 exhibited selectivity toward HCT-116 cells (Half maximal inhibitory concentration, IC50 = 23.11 ± 1.03 µM). In zebrafish embryos, ACS03 induced an increase in lactate dehydrogenase, glutathione S-transferase, and acetylcholinesterase activities. The LD50 (lethal dose 50%) value in mice was estimated to be higher than 5000 mg/kg (intraperitoneally). In vivo, ACS03 (12.5 mg/kg) induced a significant reduction in tumor volume and cell viability. In vivo antitumor activity was associated with the nitric oxide cytotoxic effect. In conclusion, significant antitumor activity and weak toxicity were recorded for this hybrid compound, characterizing it as a potential anticancer compound.

## 1. Introduction

Cancer in the broader sense refers to more than 277 different types of cancer disease [1] and is a leading cause of death and disability, with 18.1 million people diagnosed globally and more than 9.5 million deaths in 2018 [2]. Unfortunately, it shows disease variety at the tissue level, and this is a major challenge for its specific diagnosis and subsequent efficacy of treatment [3,4].

Acridines are interesting heterocyclic structures that are much-sought-after targets due to their broad biological properties since they are used as antitumor, antiparasitic, and antibacterial agents. For decades, they have been widely explored in the field of anticancer therapeutics, especially against leukemia [5,6]. These compounds, including amsacrine (m-AMSA), exhibit DNA-intercalating and topoisomerase-inhibiting activity [7,8].

Thiophene constitutes a five-membered heterocyclic scaffold that contains sulfur, and it has attracted attention because of its presence in some marketed drugs including antiasthma, diuretic, antihistaminic, and anticancer drugs [9,10]. Thiophene compounds have been shown to exhibit cytotoxicity in several cancer cell lines [11,12]. The literature shows that these compounds may induce cell cycle arrest and apoptosis and affect tubulin polymerization [13].

Nevertheless, the clinical usefulness of acridine and thiophene compounds is limited due to the risk of high toxicity and drug resistance [14,15]. Moreover, the research and development of new alternatives for cancer treatment is necessary to provide a greater response to the tumor with less toxicity. The strategy of molecular hybridization has potential for the planning and development of new drugs. This technique is based on the combination of specific structural characteristics of different bioactive moieties to produce new compounds which may display better target affinity and biological efficacy, a modified selectivity profile, and less serious side effects, when compared to its precursors [5]. 

**Scheme 1:**
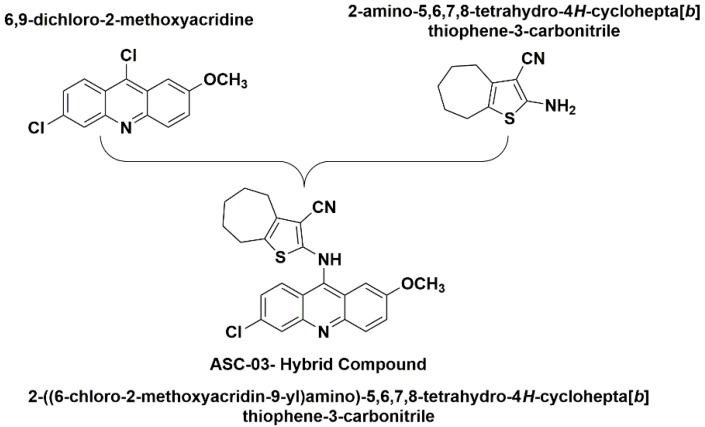
Structures of 6,9-diclhoro-2-methoxyacridine, 2-amino-5,6,7,8-tetrahydro-4H-cyclohepta[b]thiophene-3-carbonitrile, and 2-((6-Chloro-2-methoxyacridin-9-yl)amino)-5,6,7,8-tetrahydro-4H-cyclohepta[b]-thiophene-3-carbonitrile (ACS03).

A new hybrid thiophene–acridine, 2-((6-Chloro-2-methoxyacridin-9-yl)amino)-5,6,7,8-tetrahydro-4H-cyclohepta[b]-thiophene-3-carbonitrile (ACS03, Scheme 1), was previously synthesized. This compound showed antileishmanial activity and no cytotoxicity against erythrocytes. Additionally, other hybrid thiophene–acridine compounds have been characterized with the ability to interact with Leishmania DNA [16]. Then, considering the pharmacological potential of acridine and thiophene compounds, the present study aimed to investigate the toxicity and antitumor effect of the hybrid ACS03 on in vitro and in vivo models. 

## 2. Results

### 2.1. Cytotoxicity Assay

ACS03 exhibited selectivity toward HCT-116 cells (human colon carcinoma cell line; IC50 = 23.11 ± 1.03 µM), whereas no cytotoxicity was observed for other cell lines (IC50 > 50 µM). Doxorubicin was the positive control, with IC50 = 2.59 µM on HCT-116 cells. For the HaCat non-tumor cell line and peripheral blood mononuclear cells (PBMC), a lesser cytotoxic effect was observed (IC50 = 62.18 ± 1.15 µM and 115.2 ± 5.82 µM, respectively) compared to HCT-116 cells (Table 1). 

In addition, the cytotoxic effect of the ACS03 precursors was evaluated against HCT-116 cells. Higher cytotoxic activity of ACS03 compared to its precursors 2-amino-thiophene (IC50 > 1000 µM) and 6,9-diclhoro-2-methoxi-acridine (IC50 = 202.3 ± 1.19 µM) was observed.

### 2.2. Toxicity in Zebrafish Embryos

The toxicological evaluation in zebrafish embryos revealed that ACS03 did not present lethal effects during embryonic and larval development (LC50 higher than 1000 µM). In the morphological analysis of embryos, it was observed that ACS03 induced morphological changes in zebrafish embryos only at higher concentrations, such as spinal deformation (EC50 = 171.96 µM), reduction in larval length (EC50 = 126.42 µM), and pericardial edema (EC50 > 1000 µM).

### 2.3. Biochemical Biomarkers in Zebrafish Embryos

**Figure 1:**
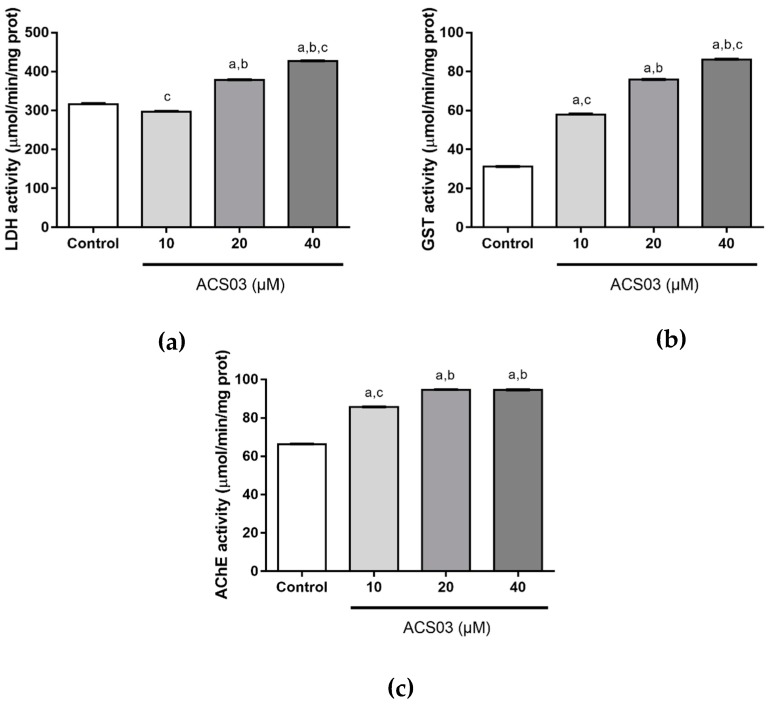
Biomarker activities (mean value ± standard error) in zebrafish larvae after 96 h exposure to different concentrations of ACS03 (*n* = 4). (**a**) Lactate dehydrogenase (LDH) activity; (**b**) Glutathione S-transferase (GST) activity; (**c**) Acetylcholinesterase (AChE) activity. ^a^ *p* < 0.05 compared to control group, ^b^ *p* < 0.05 compared to ACS03 10 µM. ^c^ *p* < 0.05 compared to ACS03 20 µM (one-way ANOVA followed by Tukey’s multiple comparison test, *p* < 0.05).

The effects of ACS03 on the biomarker activities of LDH (Lactate dehydrogenase), GST (Glutathione S-transferase), and AChE (Acetylcholinesterase) in embryos are presented in Figure 1. ACS03 induced a significant increase in LDH activity (378.44 ± 0.58 and 426.74 ± 0.79 at 20 and 40 µM, respectively; *p* < 0.05) compared to the control group (316.6 ± 1.28). In GST activity evaluation, an increase in a dose-dependent manner was observed (57.96 ± 0.23, 75.83 ± 0.13, and 86.14 ± 0.21 at 10, 20, and 40 µM ACS03, respectively; *p* < 0.05 compared to the control group, 31.8 ± 0.05). In addition, ACS03 induced an increase in AChE activity at all doses tested (85.67 ± 0.11, 94.59 ± 0.20, and 94.54 ± 0.10 at 10, 20, and 40 µM ACS03, respectively; *p* < 0.05 compared to the control group, 66.35 ± 0.04).

### 2.4. Acute Preclinical Toxicity

In the acute toxicity assay, no death was observed after the 300 mg/kg ACS03 treatment. Following Organization for Economic Co-operation and Development (OECD) guideline no. 423, the next step was to repeat the test. Again, no death was recorded. A new experiment was carried out twice with a dose of 2000 mg/kg. ACS03 did not induce death in any of the experimental animals. The LD50 (lethal dose 50%) value was estimated to be higher than 5000 mg/kg.

### 2.5. In Vivo Antitumor Activity

**Figure 2:**
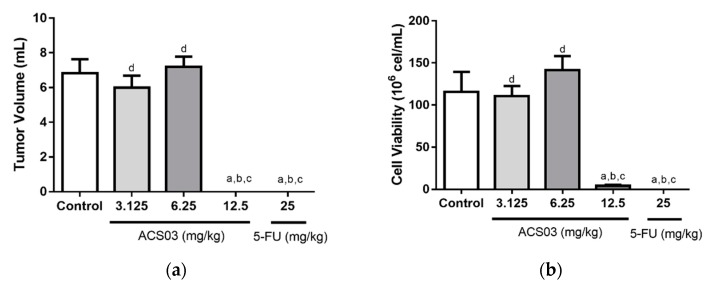
Effect of ACS03 (3.125, 6.25, or 12.5 mg/kg) on (**a**) tumoral volume and (**b**) cell viability. Data are presented as mean ± standard error of mean (SEM) of six animals analyzed by analysis of variance (ANOVA) followed by Tukey’s test. ^a^ *p* < 0.05 compared to tumor control group, ^b^ *p* < 0.05 compared to 3.125 mg/kg dose. ^c^ *p* < 0.05 compared to compared to 6.25 mg/kg dose. ^d^ *p* < 0.05 compared to compared to 12.5 mg/kg dose.

ACS03 (12.5 mg/kg) significantly reduced tumor volume (0.0 mL; *p* < 0.05) and cell viability (4.12 ± 1.28 × 10^6^ cells/mL; *p* < 0.05), compared to the control group (6.93 ± 0.79 mL and 115.5 ± 23.97 × 10^6^ cells/mL, respectively) (Figure 2). Similar results were observed for 5-fluorouracil (5-FU).

### 2.6. Quantification of Nitrite Levels

**Figure 3:**
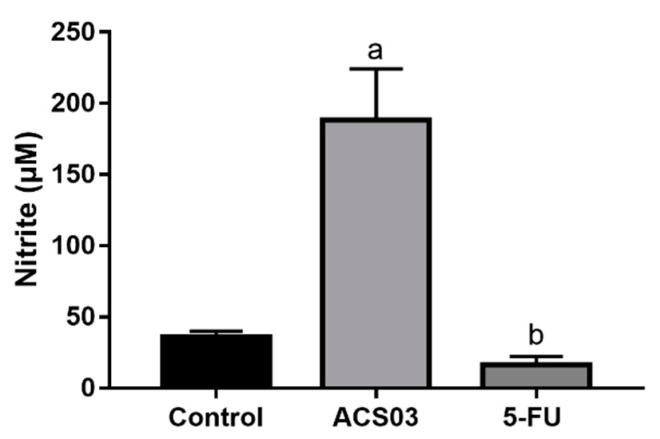
Effect of ACS03 (12.5 mg/kg) and 5-FU (25 mg/kg) on quantification of nitrite in the peritoneal fluid of Ehrlich ascites carcinoma transplanted mice. Data are presented as mean ± SEM of six animals analyzed by analysis of variance (ANOVA) followed by Tukey’s test. ^a^ *p* < 0.05 compared to tumor control group. ^b^ *p* < 0.05 compared to ACS03.

ACS03 (12.5 mg/kg) induced a significant increase in nitrite concentration (188.5 ± 7.98 µM; *p* < 0.05) as compared to the control group (36.09 ± 0.72 µM) (Figure 3). 5-FU (25 mg/kg) induced no significant change in nitrite concentration (16.3 ± 2.91 µM). 

## 3. Discussion

The discovery of novel anticancer drugs is critical for pharmaceutical research and development and patient treatment [17]. In this study, we investigated the in vitro and in vivo anticancer activity and toxicity of a new thiophene–acridine hybrid, ACS03. 

The in vitro antitumor activity of ACS03 was evaluated against different human tumor cell lines. Our data show that ACS03 has a selective effect against colorectal carcinoma cells HCT-116. Colorectal carcinoma is the second leading cause of cancer-related deaths in the world and the third most common cause of cancer-related death in the United States [18,19]. Therefore, it is indispensable to discover new compounds for its prevention and treatment [20]. Literature reports have shown the antitumor activity of acridine and thiophene compounds against colorectal carcinoma cells [6,21]. However, the precursor acridine and thiophene compounds were also tested against HCT-116 cells, and very low cytotoxicity was observed. These results indicate that hybridization of the thiophene and acridine nuclei was effective in increasing the antitumor activity above that of the separate compounds. This is the first time that a hybrid thiophene–acridine compound with antitumor activity has been described. 

The clinical use of acridine and thiophene compounds is limited by problems such as side effects, drug resistance, and low bioavailability, which have encouraged further modifications of these compounds [14,22]. Previous data have shown cytotoxicity of acridine compounds, including amsacrine, against peripheral blood mononuclear cells (PBMC) [23]. Similarly, the high toxicity described for thiophene compounds against normal cells has limited their utility [24]. Herein, the hybrid ACS03 produced low cytotoxicity against non-tumor HaCat and PBMC cells, suggesting that the balance between antitumor activity and toxicity is favorable for its therapeutic application.

Embryonic and larval *Danio rerio* (zebrafish) have previously been used as a toxicological model to conduct rapid tests and developmental toxicity assays, including toxicity assessment of pharmaceutical compounds [25]. Fortunately, many toxic responses have been conserved between zebrafish and human toxicological studies [26,27,28]. Therefore, zebrafish embryos are valuable in the assessment of system toxicity, which can be evaluated using overall embryonic mortality as a measure from which the working range of a compound can be determined [29]. In this study, we showed that ACS03 did not induce mortality of fish embryos and larvae; however, the embryos presented some morphological changes like spinal deformation, reduction in larval length, and pericardial edema. Nevertheless, these morphological changes occurred at concentrations 5 times higher than the IC50 of ACS03 on HCT-116 cells. Several reports also support the idea that zebrafish is a very sensitive model of toxicity evaluation, and early stages are commonly the most sensitive to chemical exposure [30]. 

We also analyzed the enzymatic activity in zebrafish larvae (lactate dehydrogenase (LDH), glutathione S-transferase (GST), and acetylcholinesterase (AChE) activities). LDH is a metabolic enzyme involved in the anaerobic pathway of carbohydrate metabolism, and it has been used as a biomarker of stress in fish [31]. ACS03 increased the LDH activity, showing that there was a high energy requirement in zebrafish larvae exposed to the treatment. AChE is responsible for the enzymatic degradation of acetylcholine (ACh), and its absence or low activity causes high levels of systemic ACh, which acts as a neurotransmitter [32]. Herein, we observed an increase in AChE activity, which is in general associated to neurotoxicity [33]; however, the literature also suggests that AChE may display antitumor activity, in addition to correlating tumor malignancy with low activity of AChE [34,35,36]. GSTs are vital in the Phase II detoxification enzymes pathway in humans, and they provide protection against toxins by catalyzing toxin conjugation with Glutathione (GSH) or passively binding to various exogenous/endogenous toxic molecules, including environmental toxins, carcinogens, chemotherapeutic agents, and products of oxidative stress [37,38]. We showed an increase in GST activity in larvae exposed to ACS03; this may be associated with antioxidant capacity increase.

In vivo preclinical toxicological analysis was performed in mice to determine safe doses to be used for pharmacological tests. Considering the high LD50 (5000 mg/kg) estimated, ACS03 has low toxicity. If the LD50 of the test substance is 3 times more than the minimum effective dose, the substance is considered a good candidate for further studies [39].

To evaluate the in vivo antitumor effect of ACS03, we used the Ehrlich ascites carcinoma (EAC) model, a spontaneous murine mammary adenocarcinoma that is a well-established model for testing new drugs [40,41]. ACS03 reduced the tumor volume and cell viability, indicating significant in vivo activity of this compound. Our data corroborate the literature results of separate acridine and thiophene compounds showing an antitumor effect on the Ehrlich ascites carcinoma model [42,43].

Oxide nitric (NO) is a ubiquitous, water-soluble, free radical gas which plays a key role in various physiological and pathological processes, including cancer. Literature reports have indicated that NO may have dual effects in cancer [44]: At low concentration, NO causes tumor progression in antiapoptotic, pro-growth, and proangiogenic ways. On the other hand, at high concentrations, NO has a potential role in cancer therapy by inducing proapoptotic and antiangiogenic effects and inhibition of proliferation [45]. In the Ehrlich ascites carcinoma model, we demonstrated that ACS03 treatment induced a significant increase in nitrite levels, which is associated with increased NO levels, suggesting that the ACS03 mechanism of antitumor action involves NO cytotoxic effects. 

In conclusion, our findings suggest that thiophene–acridine hybrid ACS03 has potential antitumor activity and low toxicity, and it has potential as an anticancer compound.

## 4. Materials and Methods 

### 4.1. Treatment

ACS03 was provided by Dr. Ricardo Olimpio de Moura from the Laboratory of Synthesis and Vectorization of Molecules (LSVM) of the State University of Paraíba (UEPB), João Pessoa, Brazil, and it was synthesized as described previously [16]. For in vitro assays, ACS03 was dissolved in 100% DMSO and added to the culture medium at a final concentration of 0.5%. For in vivo assay, ACS03 was dissolved in 12% (v/v) Tween 80 in saline.

### 4.2. Cell Lines

Colorectal carcinoma cells (HCT-116), chronic myelogenous leukemia (K562), and acute promyelocytic leukemia (HL-60) were generously provided by Dr. Manoel de Moraes (Federal University of Ceará, Fortaleza, Brazil) and cultured in Roswell Park Memorial Institute 1640 (RPMI 1640; Sigma Aldrich, St. Louis, MO, USA) medium supplemented with 10% fetal bovine serum (GIBCO, Grand Island, NY, USA) and 1% penicillin–streptomycin (Sigma Aldrich, St. Louis, MO, USA). Human keratinocyte cells (HaCaT; provided by Dr. Jaciana Aguiar from Federal University of Pernambuco, Recife, Pernambuco, Brazil), cervical cancer cells (HeLa; purchased from the cell bank of Rio de Janeiro, Rio de Janeiro, Brazil), and breast cancer cells (MCF-7; provided by Dr. João Ernesto de Carvalho from University of Campinas, Campinas, São Paulo, Brazil) were grown and maintained in Dulbecco’s Modified Eagle’s Medium (DMEM; Sigma Aldrich, St. Louis, MO, USA) supplemented with 10% fetal bovine serum (GIBCO, Grand Island, NY, USA) and 1% penicillin–streptomycin (Sigma Aldrich, St. Louis, MO, USA). All cell types were maintained in 5% CO_2_ at 37 °C with humidity.

Ehrlich ascites carcinoma was generously provided by Pharmacology and Toxicology Division, CPQBA, UNICAMP (Paulínia, São Paulo, Brazil). The cells were maintained in the peritoneal cavities of Swiss mice in the Dr. Thomas George Bioterium (Research Institute in Drugs and Medicines/Federal University of Paraíba, João Pessoa, Brazil).

### 4.3. Peripheral Blood Mononuclear Cells (PBMC)

Heparinized blood was collected from healthy donors, and the PBMC were isolated by a standard method of density-gradient centrifugation using Ficoll-Histopaque (Sigma Aldrich, St. Louis, MO, USA). PBMC were cultivated in RPMI-1640 medium supplemented with 10% fetal bovine serum, 1% penicillin–streptomycin, and 2% phytohaemagglutinin. Cells were kept at 37 °C with 5% CO_2_ for 24 h before the start of the experiment. This study was approved by the Ethics Committee of the Human Health Sciences Center Research (CCS), UFPB (CAAE: 22986519.0.0000.5188), Brazil.

### 4.4. Zebrafish Embryos

The zebrafish (*Danio rerio*) embryos were provided by the zebrafish facility established at Department of Molecular Biology, Federal University of Paraíba (João Pessoa, Brazil). To obtain embryos, an egg trap was placed overnight in a tank containing male and female specimens (1:1 ratio) of a wild-type strain one day prior to testing. One hour after the beginning of the light cycle (14 h light/10 h dark), eggs were collected and rinsed with E3 medium (5 mM NaCl, 0.17 mM KCl, 0.33 mM CaCl_2_, and 0.33 mM MgSO_4_). Viable fertilized eggs were selected using a stereomicroscope (80× magnification), and embryotoxicity analysis was carried out. All experiments were approved by the Committee on Ethical Use of Animals in Research (CEUA) of Federal University of Paraíba, Brazil, certified by number 5900310718/2018.

### 4.5. Animals

Female Swiss albino mice (*Mus musculus*) weighing between 28 and 32 g, with an approximate age of 60 days and obtained from the Dr. Thomas George Bioterium (Research Institute in Drugs and Medicines/Federal University of Paraíba, Paraíba, Brazil) were used. The animals were grouped in polyethylene cages containing six animals, with free access to food (Purina^®^ feed pellets, St. Louis, MO, USA) and water. The animals were kept under controlled conditions (21 ± 1 °C, 12 h/12 h off light/dark cycle). All experiments were approved by the Committee on Ethical Use of Animals in Research (CEUA) of Federal University of Paraíba, Brazil, certified by number 093/2017.

### 4.6. In Vitro Assay

#### Cell Viability Assay

MTT (3-(4,5-dimethylthiazol-2-yl)-2,5-diphenyltetrazolium bromide) reduction assay was used to evaluate the cytotoxicity of ACS03 and its precursors (2-amino-5,6,7,8-tetrahydro-4H-cyclohepta[b]thiophene-3-carbonitrile and 6,9-dichloro-2-methoxyacridine) against tumor and non-tumor cell lines and PBMC. For the MTT assay, cell lines (3 × 10^5^ cells/mL) and PBMC (1 × 10^6^ cells/mL) were seeded on 96-well plates and cultured for 24 h. ACS03 (3.125–200 µM) was added to the cell cultures and incubated for 72 h. After incubation, 110 µL of the supernatant was removed and 10 μL of the MTT reagent (Sigma Aldrich, St. Louis, MO, USA) was added to each well, and the plates were then incubated at 37 °C. Following 3 h incubation, 100 μL of sodium dodecyl sulfate (SDS) was added to dissolve the formazan crystals produced and left overnight to incubate [46]. The amount of formazan is proportional to the number of viable cells present. The optical density was measured using a microplate spectrophotometer reader (Microplate reader BioTek Instruments, Sinergy HT, Winooski, VT, USA) at *λ* = 570 nm and used to calculate the IC50 (half maximal inhibitory concentration).

### 4.7. In Vivo Assays

#### 4.7.1. Acute Toxicity Test Using Zebrafish Embryos

The fish embryo acute toxicity (FET) test was conducted with ACS03 according to OECD’s guideline no. 236, with slight modifications [47]. Zebrafish embryos of up to 3 hpf (hours post fertilization) of age were initially exposed to 250–15.62 µM. For each concentration tested, we prepared a 96-well plate containing 20 fertilized eggs (1 embryo per well) exposed to the test sample and 4 embryos exposed only to E3 medium (internal controls). The exposure was performed for 96 h, and the embryos were analyzed every 24 h for the apical endpoints: egg coagulation; lack of somite formation; lack of detachment of the tail-bud from the yolk sac; and lack of heartbeat. In the presence of any of these endpoints, the embryo/larva was considered dead. The number of deaths was used to calculate the LC50 (median lethal concentration). Sublethal effects were also recorded in order to obtain the EC50 (median effect concentration), them being spinal deformation, reduction in larval length, and pericardial edema. The exposures were under static conditions (without renovation of test sample or E3 medium). Observations were performed in a stereomicroscope (80× magnification) and photographed (Zeiss). After 96 h, surviving larvae were euthanized with eugenol and properly discarded.

#### 4.7.2. Enzyme Testing Using Zebrafish Larvae

The FET test was repeated for ACS03 under the same conditions described above, but this time the embryos were independently exposed to three concentrations (10, 20, and 40 µM). At the end of the exposure period (96 h), the larvae were quickly frozen in a ratio of 1 larva per 100 µL of 0.1 M sodium phosphate buffer, pH 7.4.

Larval groups from each of the three tested concentrations and a negative control group (exposed to E3 medium only) were macerated using 1:9 (w/v) cold 0.9% NaCl solution. The homogenates were centrifuged at 10,000× *g* for 10 min at 4 °C, and the resulting supernatants were used for the measurement of soluble protein content and enzyme activity. The activities of acetylcholinesterase (AChE), glutathione S-transferase (GST), and lactate dehydrogenase (LDH) were measured according to Domingues et al. (2010) [48]. Tests were done in quadruplicate for each enzyme.

#### 4.7.3. Assessment of Acute Preclinical Toxicity in Mice

The acute toxicity test in mice was performed according to the Guideline for Testing of Chemicals no. 423 of the Organization for Economic Co-operation and Development (OECD) [49]. Mice were divided into different groups (*n* = 3 females/group). The control group received vehicle alone (12% (v/v) Tween 80 in saline), and ACS03 was tested in single doses of 300 or 2000 mg/kg, intraperitoneally (i.p.). The dose responsible for the death of 50% of the experimental animals (LD50) was estimated. 

#### 4.7.4. Evaluation of In Vivo Antitumor Activity in the Ehrlich Ascites Carcinoma Model

Mice were inoculated with 4 × 10^6^ cells/mL of Ehrlich tumor cells (0.5 mL/animal), intraperitoneally (i.p.) [50]. After 24 h, mice were divided into five groups (*n* = 6/group) and treated with vehicle alone (12% (v/v) Tween 80 in saline), 5-FU (standard drug, 25 mg/kg), or ACS03 (3.125, 6.25, 12.5, or 25 mg/kg) for seven consecutive days (i.p.). On the ninth day, all animals were anesthetized with ketamine hydrochloride (100 mg/kg), intramuscularly (i.m.) and xylazine hydrochloride (16 mg/k) intraperitoneally (i.p.) and were euthanized by cervical dislocation. The volume of ascitic fluid was collected from the peritoneal cavity to determine the tumor volume (mL). Trypan blue assay was used to evaluate the cell viability [51].

#### 4.7.5. Quantification of Nitrite Levels in the Ehrlich Ascites Carcinoma Model

The nitrite concentration in the ascitic fluid was measured using Griess reagent [52]. For this, the peritoneal fluid of control and treated animals (12.5 mg/kg ACS03 and 25 mg/kg 5-FU) was used. The optical density was measured using a microplate spectrophotometer reader (Microplate reader BioTek Instruments, Sinergy HT, Winooski, VT, USA) at *λ* = 560 nm. Concentrations of nitrite were calculated from a standard curve previously established with known molar concentrations of sodium nitrite. The tests were done in quadruplicate and the values expressed in µM [51].

### 4.8. Statistical Analysis

The results are expressed as the mean ± SEM. Statistical analysis of data was performed using analysis of variance (ANOVA), followed by Tukey’s test (*p* < 0.05). For zebrafish embryo cytotoxicity, the median lethal concentration (LC50) values and the median effect concentration (EC50) values were calculated by probit regression analysis [53].

## Figures and Tables

**Table 1:** Cytotoxicity of ACS03 against various cell types by MTT (3-(4,5-dimethylthiazol-2-yl)-2,5-diphenyltetrazolium bromide) assay. Mean IC50 (half maximal inhibitory concentration) values were calculated from cell growth curves (*n* = 3).

Cell Lines *	ACS03 IC50 (µM)
**HCT-116**	23.11 ± 1.03
**HeLa**	>50
**MCF-7**	>50
**K562**	>50
**HL-60**	>50
**HaCat**	62.18 ± 1.15 ^a^
**PBMC**	115.2 ± 5.82 ^a^

^a^ Statistically significantly different from ACS03 treatment against HCT-116 cells (*p* < 0.05). Statistical analysis was performed using one-way ANOVA followed by Tukey’s test. * HCT-116: human colon carcinoma cell line; HeLa: human cervical cancer cell line; MCF-7: breast cancer cell line; K562: chronic myeloid leukemic cell line; HL-60: human promyelocytic leukemia cell line; HaCat: Human immortalized keratinocytes cell line; PBMC: peripheral blood mononuclear cells.
